# *In Vivo* Antigen Expression Regulates CD4 T Cell Differentiation and Vaccine Efficacy against Mycobacterium tuberculosis Infection

**DOI:** 10.1128/mBio.00226-21

**Published:** 2021-04-20

**Authors:** Helena Strand Clemmensen, Jean-Yves Dube, Fiona McIntosh, Ida Rosenkrands, Gregers Jungersen, Claus Aagaard, Peter Andersen, Marcel A. Behr, Rasmus Mortensen

**Affiliations:** a Department of Infectious Disease Immunology, Statens Serum Institut, Copenhagen, Denmark; b Department of Health Technology, Technical University of Denmark, Kongens Lyngby, Denmark; c Department of Immunology and Microbiology, University of Copenhagen, Copenhagen, Denmark; d Department of Microbiology and Immunology, McGill University, Montréal, Canada; e Infectious Diseases and Immunity in Global Health Program, Research Institute of the McGill University Health Centre, Montréal, Canada; f McGill International TB Centre, Montréal, Canada; g Department of Medicine, McGill University Health Centre, Montréal, Canada; National Institute of Allergy and Infectious Diseases

**Keywords:** vaccination, MPT70, ESAT-6, immunization, *in vivo* expression, *Mycobacterium tuberculosis*, T cell differentiation

## Abstract

New vaccines are urgently needed against Mycobacterium tuberculosis (Mtb), which kills more than 1.4 million people each year. CD4 T cell differentiation is a key determinant of protective immunity against Mtb, but it is not fully understood how host-pathogen interactions shape individual antigen-specific T cell populations and their protective capacity. Here, we investigated the immunodominant Mtb antigen, MPT70, which is upregulated in response to gamma interferon (IFN-γ) or nutrient/oxygen deprivation of *in vitro*-infected macrophages. Using a murine aerosol infection model, we compared the *in vivo* expression kinetics of MPT70 to a constitutively expressed antigen, ESAT-6, and analyzed their corresponding CD4 T cell phenotype and vaccine protection. For wild-type Mtb, we found that *in vivo* expression of MPT70 was delayed compared to ESAT-6. This delayed expression was associated with induction of less differentiated MPT70-specific CD4 T cells but, compared to ESAT-6, also reduced protection after vaccination. In contrast, infection with an MPT70-overexpressing Mtb strain promoted highly differentiated KLRG1^+^CX3CR1^+^ CD4 T cells with limited lung-homing capacity. Importantly, this differentiated phenotype could be prevented by vaccination, and against the overexpressing strain, vaccination with MPT70 conferred protection similar to vaccination with ESAT-6. Together, our data indicate that high *in vivo* antigen expression drives T cells toward terminal differentiation and that targeted vaccination with adjuvanted protein can counteract this phenomenon by maintaining T cells in a protective less differentiated state. These observations shed new light on host-pathogen interactions and provide guidance on how future Mtb vaccines can be designed to tip the immune balance in favor of the host.

## Abstract (precis)

Tuberculosis, caused by Mtb, constitutes a global health crisis of massive proportions, and the impact of the current coronavirus disease 2019 (COVID-19) pandemic is expected to cause a rise in tuberculosis-related deaths. Improved vaccines are therefore needed more than ever, but a lack of knowledge on protective immunity hampers their development.

## INTRODUCTION

Mycobacterium tuberculosis (Mtb) has successfully survived in the human host for thousands of years and still causes 1.4 million deaths from tuberculosis (TB) disease annually (1). Formation of the granuloma is associated with the containment of infection, as the environment within the granuloma suppresses Mtb growth in multiple ways, including oxygen and nutrient deprivation, exposure to acidic pH, and production of endogenous nitric oxide. In response to this, Mtb adapts by shifting between metabolic states often characterized by alterations in gene expression and thus changes in protein secretion and antigenic repertoire (2–5).

As part of evolutionary adaptation, virulent strains across the mycobacterial tuberculosis complex (MTBC) vary in their expression of certain antigens, like the major secreted immunogenic protein 70 (MPT70) (6). Mtb produces very small amounts of MPT70 in *in vitro* cultures (7), but multiple studies have demonstrated that gamma interferon (IFN-γ) activation (5, 6) or starvation (8) induces MPT70 expression upon *in vitro* infection. Although MPT70 is a well-known immunodominant antigen during Mtb infection in both mice and humans (9–13), nothing is known about how MPT70’s *in vivo* antigen expression profile relates to the T cell phenotype it induces and its protective capacity as a vaccine antigen (13–16).

CD4 T cells are essential for protective immunity against Mtb (17–20), and there is mounting evidence that CD4 T cells develop into terminally differentiated IFN-γ-producing effector T cells upon continuous antigen stimulation (21–23). The development of effector CD4 T cells is linked to sustained expression of markers and chemokine receptors associated with terminal Th1 differentiation and poor lung homing (24, 25). Less differentiated CXCR3^+^T-bet^dim^ CD4 T cells are able to enter the lung parenchyma and inhibit Mtb growth (25, 26), while terminally differentiated CD4 T cells coexpressing CX3CR1 and KLRG1 accumulate in the lung vasculature and provide no pulmonary control of Mtb infection (27, 28). Individual differences in antigen expression are suggested to shape T cell phenotype (22) and may therefore be a key determinant of vaccine protection.

The goal of this study was to investigate the impact of *in vivo* antigen expression on antigen recognition kinetics and adaptive immunity during Mtb infection, with MPT70 as a unique tool. Using the well-described 6-kDa early secretory antigenic target (ESAT-6) as a prototypic immunodominant model antigen (22, 29–31), we show that MPT70 displays delayed *in vivo* antigen expression as well as delayed immune recognition. This is associated with the induction of less differentiated CD4 T cells but also lower protection in mice vaccinated with MPT70. On the basis of these observations, we hypothesize that high constitutive antigen expression is associated with increased T cell differentiation but also improved vaccine capacity. In support of this, we demonstrate that artificial overexpression of MPT70 leads to accelerated CD4 T cell differentiation and diminished lung-homing capacity. However, vaccination with MPT70 counteracts this by stabilizing a low degree of T cell differentiation and increases protection substantially in the MPT70-overexpressing strain compared to wild-type (WT) Mtb. Our study therefore reveals that antigen expression kinetics regulates CD4 T cell differentiation during infection and establishes a link between *in vivo* antigen expression, T cell differentiation, and vaccine protective capacity. This has implications for rational vaccine design, and future efforts in TB antigen discovery might use antigen-specific T cell differentiation as a readily measurable proxy for high *in vivo* antigen expression and increased vaccine potential.

## RESULTS

### Delayed *in vivo* antigen transcription results in late immune recognition of MPT70.

Previous studies indicate that MPT70 expression by Mtb is very low during *in vitro* cultivation (7, 11) but that expression is induced upon IFN-γ activation (5, 6) or nutrient deprivation (8). On the basis of these studies, we hypothesized that *in vivo* transcription and immune recognition of MPT70 would be delayed compared to ESAT-6, a constitutively expressed virulence factor (32, 33).

**FIG 1:**
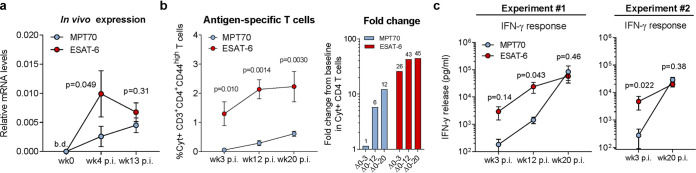
*In vivo* antigen expression and immune recognition of MPT70 are delayed during M. tuberculosis (Mtb) Erdman infection. CB6F1 mice were infected by the aerosol route with Mtb Erdman. (a) MPT70 and ESAT-6 *in vivo* gene expression were assessed preinfection (week 0 [wk0]) and 4 and 13 weeks postinfection (p.i.) (*n* = 4). The expression preinfection was below detection levels (b.d.). Values shown are means ± standard errors of the means (SEM) (error bars). A two-tailed, paired *t* test was used to assess statistical differences. (b, left) At weeks 3, 12, and 20 after Mtb infection, lungs were harvested for immunological analyses. The frequency of cytokine-producing CD3^+^ CD4^+^ T cells specific for MPT70 and ESAT-6 for the same time points as shown in panel c, experiment 1 (medium cytokine production subtracted) analyzed by flow cytometry using the antibodies shown in Table 3 (*n* = 4). Values shown are means ± SEM. One-way analysis of variance (ANOVA) with Tukey’s multiple-comparison test was used to assess statistical differences. (b, right) Fold change in cytokine-producing CD4 T cells from baseline. (c) Lung cells from infected mice were restimulated *in vitro* with media, MPT70, or ESAT-6 for 5 days. Culture supernatant was harvested and measured for IFN-γ levels in two individual experiments (*n* = 4). Values were log transformed and shown as means ± SEM. A two-tailed, paired *t* test was used to assess statistical differences.

To map the kinetics of MPT70 expression *in vivo*, we infected a group of CB6F1 mice with Mtb Erdman, which we expected to produce small amounts of MPT70 (7). RNA was extracted from the postcaval lobe, and cDNA was quantified by real-time quantitative PCR (qPCR) using dually labeled probes and normalized to 16S rRNA. Expression levels of MPT70 and ESAT-6 mRNA were analyzed prior to infection (week 0), at an early time point (week 4), and at a late time point (week 13). As expected, expression levels were below detection level prior to infection (Fig. 1a). At week 4, MPT70 expression was low and significantly lower than ESAT-6, but as the infection progressed to week 13, MPT70 expression increased and approached levels of ESAT-6, indicating a delayed expression profile (Fig. 1a).

We next investigated the kinetics of the immune recognition to the two antigens during the course of infection. Mice were infected as previously and antigen-specific immune responses were detected at 3, 12, and 20 weeks postinfection, either by intracellular cytokine staining (ICS) measuring the frequency of antigen-specific CD4 T cells producing interleukin 2 (IL-2), tumor necrosis factor alpha (TNF-α), or IFN-γ (Fig. 1b; see also Fig. S1 in the supplemental material) or IFN-γ release in cultures of stimulated splenocytes (Fig. 1c). Notably, in two independent experiments, we observed that the immune recognition of MPT70 was very low in the early phase of infection but continued to increase as the infection progressed to week 12 and 20 (Fig. 1b and c). This was in contrast to ESAT-6 responses that were greater at week 3 and 12, after which they plateaued.

10.1128/mBio.00226-21.1 FIG S1 Gating strategy for antigen-specific CD4 T cells after intracellular staining (ICS). (a) Spleens and lungs were harvested from mice and prepared as single-cell suspensions. Representative gating from sample WT H37Rv, ESAT-6-specific T cells 3 weeks after Mtb infection using the antibodies shown in Table 5. Cells were gated as singlets, lymphocytes, and CD3^+^ CD4^+^ or CD8^+^ CD4-positive (CD4^+^) T cells. CD44^high^ CD4 T cells were analyzed for their intracellular production of IFN-γ, TNF-α, IL-2, and IL-17A. The frequency of antigen-specific CD4 T cells was determined by a make or gate for IFN-γ, TNF-α, IL-2, and IL-17A (i.e., T cells can produce one or more of the cytokines). T cell differentiation degrees were analyzed with a combination gate for IFN-γ, TNF-α, and IL-2, characterizing the cytokine subsets of T cells. The frequencies of CD45^+^, KLRG1, PD-1, CXCR3, and CX3CR1 were assessed on antigen-specific T cells. Fluorescence minus one (FMO) controls were used to set boundary gates for CD44, KLRG1, PD-1, CXCR3, and CX3CR1. (b, left) ESAT-6- and MPT70-specific CD4 T cells 20 weeks after Mtb infection (*n* = 4). (b, right) Frequencies of antigen-specific CD4 T cells producing IFN-γ, TNF-α, IL-2, and IL-17A (*n* = 4). Download FIG S1, TIF file, 1.4 MB. Copyright © 2021 Strand Clemmensen et al. 2021 Strand Clemmensen et al. https://creativecommons.org/licenses/by/4.0/ This content is distributed under the terms of the Creative Commons Attribution 4.0 International license.

Together, these data indicate that *in vivo* expression of MPT70 is delayed compared to ESAT-6 and that this difference in kinetics is associated with lower initial CD4 T cell responses and/or a delayed onset MPT70-specific adaptive immunity.

### MPT70-specific CD4 T cells maintain a low degree of differentiation.

**FIG 2:**
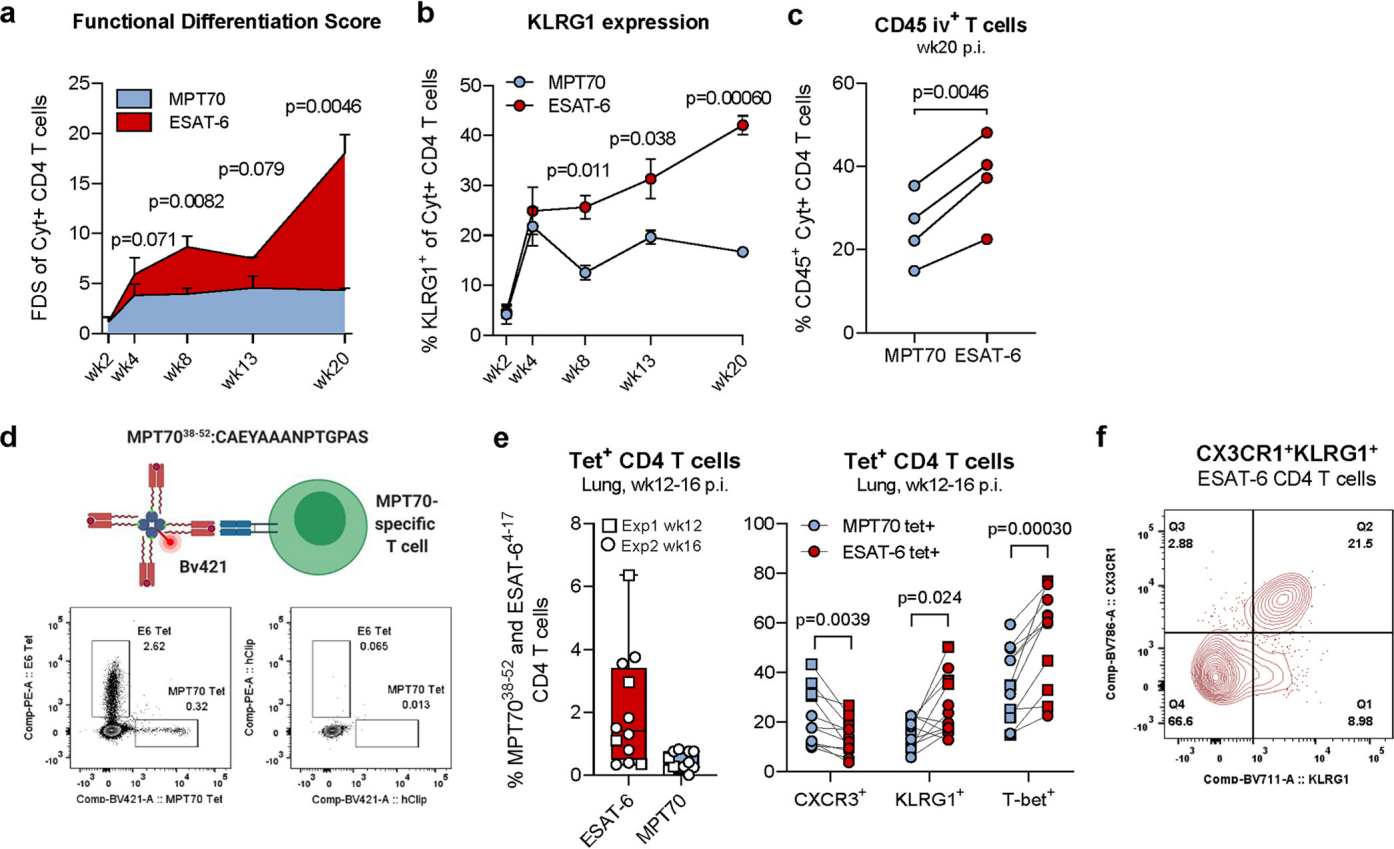
MPT70-specific CD4 T cells maintain a low differentiation state compared to ESAT-6. (a) The functional differentiation score (FDS) of MPT70- and ESAT-6-specific CD4 T cells over the course of Mtb Erdman infection (*n* = 4). The FDS is defined as the ratio of all IFN-γ-producing CD4 T cell subsets divided by subsets producing other cytokines (IL-2, TNF-α), but not IFN-γ (high FDS = high IFN-γ production). Multiple *t* tests with correction for multiple testing using the Holm-Sidak method were used to assess statistical differences. Values shown are means ± SEM. Flow cytometry gating is shown as depicted in Fig. S1a in the supplemental material, using the antibodies shown in Table 4. (b) Frequencies of KLRG1-expressing MPT70- and ESAT-6-specific CD4 T cells throughout infection (*n* = 4). Values shown are means ± SEM. Multiple *t* tests with correction for multiple testing using the Holm-Sidak method were used to assess statistical differences. (c) Frequency of CD45-labeled MPT70- and ESAT-6-specific CD4 T cells in the lung-associated vasculature (CD45^+^) 20 weeks postinfection (p.i.) with Mtb (*n* = 4). A two-tailed, paired *t* test was used. (d, top) Schematic representation of custom-made I-A^b^:MPT70_38-52_ MHC-II tetramer. (d, bottom) Representative concatenated FACS plots showing frequencies of I-A^b^:MPT70_38-52_ and I-Ab:ESAT-6_4-17_ tetramer (Tet)-positive CD4 T cells or corresponding hClip tetramer-positive CD4 T cells in lungs of mice 12 weeks after Mtb infection (*n* = 4). (e) Frequency of I-A^b^:MPT70_38-52_ and I-Ab:ESAT-6^4-17^ CD4 T cells 12 to 16 weeks after Mtb infection expressing CXCR3, KLRG1, and T-bet. A parametric, two-tailed, paired *t* test was used to assess statistical differences (*n* = 12). Flow cytometry gating is shown as depicted in Fig. S3 using the antibodies shown in Table 3. (f) Concatenated FACS plot of CX3CR1^+^ KLRG1^+^ coexpressing ESAT-6^4-17^ CD4 T cells (*n* = 4).

Continuous stimulation with high levels of antigen is known to drive T cells toward terminal differentiation (21–23), and we therefore explored whether the delayed antigen availability of MPT70 favored the development of less differentiated T cells during infection. In order to address this, we first characterized MPT70- and ESAT-6-specific T cells according to their expression of intracellular cytokines associated with Th1 differentiation (Fig. S1a). We observed no or little induction of IL-17-producing CD4 T cells and the responding CD4 T cells primarily expressed IFN-γ, TNF-α, or IL-2 (Fig. S1b). As previously defined (22, 34), a functional differentiation score (FDS) can be applied as a simple measure for the T cell differentiation status and is calculated as the ratio of all highly differentiated IFN-γ-producing T cell subsets divided by less differentiated T cell subsets producing other cytokines (IL-2, TNF-α). An FDS score of >1 is therefore indicative of a response with more highly differentiated T cells than less differentiated T cells. During the first 2 weeks of infection, MPT70- and ESAT-6-specific CD4 T cells displayed similar FDSs in the range of 2. From week 2 to 4, the FDS of both T cell subsets increased to 3.8 and 5.9, respectively. From week 4 and onwards, the FDS of ESAT-6 T cells continuously increased to reach 18, while the FDS of MPT70 T cells remained constant around 4, denoting that MPT70 CD4 T cells are not driven toward terminal differentiation to the same extent as ESAT-6 (Fig. 2a). In Mtb-infected mice, CXCR3^+^KLRG1^−^Tbet^dim^ T cells migrate into the lung parenchyma and control the infection (26, 28), while intravascular (iv) T cells have a high expression of KLRG1, CX3CR1, and T-bet (24). In accordance with the FDS data, we observed a substantially lower proportion of cytokine-expressing KLRG1^+^ CD4 T cells after MPT70 stimulation compared to ESAT-6, and this difference was sustained throughout the infection (Fig. 2b). Investigating the ability of these CD4 T cell subsets to enter the infected lung tissue by CD45 iv staining further supported that a smaller fraction of MPT70-specific CD4 T cells were retained in the lung-associated vasculature (CD45 iv^+^) compared to ESAT-6 CD4 T cells (Fig. 2c).

10.1128/mBio.00226-21.3 FIG S3 Phenotyping of MPT70_38-52_ and ESAT-6_4-17_ CD4 T cells during Mtb infection. Gating strategy for tetramer-positive CD4 T cells. Lung cells of vaccinated and infected mice were prepared as single-cell suspensions and analyzed by flow cytometry. Shown as representative gating for tetramer-positive CD4 T cells exemplified with mouse A6 injected with saline and infected for 16 weeks using the antibodies shown in Table 3. Cells were gated as singlets and lymphocytes. Viable CD3^+^ CD4^+^ CD44^high^ T cells were stained with either I-A^b^:MPT70_38-52_ and I-Ab:ESAT-6_4-17_ tetramer. A corresponding control tetramer, hClip, was included. Tetramer-positive CD4 T cells were further characterized for their expression of KLRG1, T-bet, and CXCR3. Fluorescence minus one (FMO) controls were used to set boundary gates for KLRG1, T-bet, and CXCR3. Download FIG S3, TIF file, 0.9 MB. Copyright © 2021 Strand Clemmensen et al. 2021 Strand Clemmensen et al. https://creativecommons.org/licenses/by/4.0/ This content is distributed under the terms of the Creative Commons Attribution 4.0 International license.

We next wanted to confirm these observations using a major histocompatibility complex class II (MHC-II) tetramer. In contrast to ICS, tetramers identify antigen-specific T cells without the risk of affecting the expression of certain markers due to *ex vivo* stimulation. We therefore epitope mapped the MPT70 protein (29) and developed a murine MHC-II tetramer specific for I-A^b^:MPT70_38-52_ (see Materials and Methods, Fig. 2d, and Fig. S2). In the lungs of mice infected with Mtb for 12 to 16 weeks, we found an average of 2.02% tetramer-positive I-A^b^:ESAT-6_4-17_ and 0.44% I-A^b^:MPT70_38-52_-specific CD4 T cells (Fig. 2e). Exploring the expression of CXCR3, KLRG1, CX3CR1, and T-bet showed that MPT70_38-52_-specific CD4 T cells expressed significantly higher levels of CXCR3 (*P* = 0.0039) and significantly lower levels of KLRG1 (*P* = 0.024) and T-bet (*P* = 0.00030) compared to ESAT-6_4-17_-specific T cells (Fig. 2e). In line with the literature, the vast majority of KLRG1^+^ T cells coexpressed CX3CR1, which is associated with vascular T cells (24, 25) (Fig. 2f), and therefore in agreement with the data obtained by CD45 iv staining.

10.1128/mBio.00226-21.2 FIG S2 Epitope mapping and design of an MPT70 tetramer. (a) Splenocytes of MPT70-vaccinated mice were *in vitro* restimulated with overlapping peptides of 15 amino acids in length for 3 days (*n* = 4). The amount of IFN-γ was measured in the culture supernatant. The dominant epitope required for binding is indicated in bold blue text, and the predicted core epitope is shown in bold black text. (b, left) The minimal epitope of the 38-53 sequence of MPT70 was investigated with various lengths of peptides in MPT70-vaccinated mice 20 weeks postinfection (*n* = 4). (b, right) Comparison of the response to medium, the chosen 38-53 epitope, and recombinant MPT70. This panel shows the same data as in left panel of panel b. Download FIG S2, TIF file, 0.4 MB. Copyright © 2021 Strand Clemmensen et al. 2021 Strand Clemmensen et al. https://creativecommons.org/licenses/by/4.0/ This content is distributed under the terms of the Creative Commons Attribution 4.0 International license.

In summary, these studies show, by both cytokine production pattern and expression of differentiation markers, that MPT70-specific CD4 T cells are maintained at a lower state of differentiation throughout infection compared to ESAT-6-induced T cells. This difference translates into a higher functional capacity of MPT70-specific CD4 T cells to migrate to infected lung tissue.

### The impact of vaccinating with MPT70 is lower than for ESAT-6.

**FIG 3:**
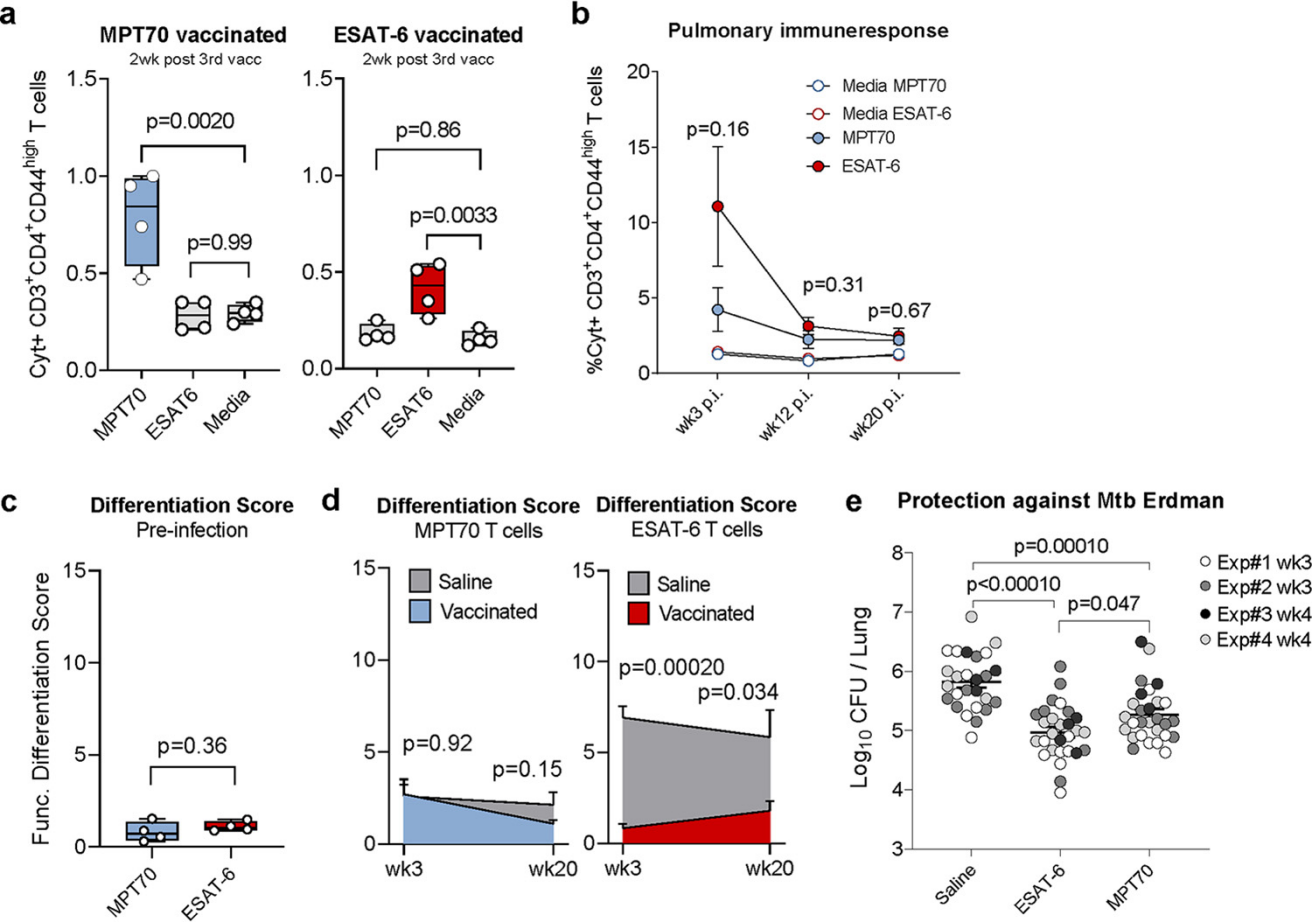
The impact of MPT70 immunization on alleviating infection-driven T cell differentiation is lower than immunization with ESAT-6. Female CB6F1 mice were immunized with either MPT70 or ESAT-6 recombinant protein three times s.c. and challenged with Mtb Erdman 6 weeks after the third immunization. (a) Frequency of MPT70- and ESAT-6-specific CD4 T cells in the spleen 2 weeks after the third vaccination (*n* = 4). Ordinary one-way ANOVA with Dunnett’s multiple-comparison test was used to assess statistical differences. (b) Frequency of MPT70- and ESAT-6-specific CD4 T cells in the lung at weeks 3, 12, and 20 after Mtb infection (*n* = 4). Values shown are means ± SEM. An unpaired *t* test was used to compare the values for ESAT-6- and MPT70-vaccinated mice. (c) Functional differentiation score (FDS) of MPT70 and ESAT-6-specific CD4 T cells preinfection in the spleen (*n* = 4). An unpaired *t* test was used to assess statistical differences. (d) FDS of MPT70- and ESAT-6-specific CD4 T cells 3 and 20 weeks after Mtb infection in the lungs of vaccinated mice and mice injected with saline (*n* = 4). Values shown are means ± SEM. An unpaired *t* test was used to compare individual time points. Flow cytometry gating is shown as depicted in Fig. S1a, using the antibodies shown in Table 4. (e) The bacterial burden was determined in the lungs of mice injected with saline or mice vaccinated with MPT70 and ESAT-6 3 to 4 weeks after Mtb infection (*n* = 26 to 28). The graph shows the results of four individual experiments (experiment 4 has already been published in reference 29). One-way ANOVA with Tukey’s multiple-comparison test was used to assess statistical differences.

The previous data showed that T cells specific for MPT70 were less differentiated than those specific for ESAT-6 during experimental infection. We next investigated the significance of this in the context of vaccine efficacy. Mice were immunized three times with MPT70 or ESAT-6 in the cationic adjuvant formulation 1 (CAF01) adjuvant (35), and immune responses were characterized 2 weeks after the final vaccination. ICS of stimulated splenocytes detected 0.79% MPT70-specific CD4 T cells compared to 0.42% for ESAT-6, showing that both antigens were immunogenic and, if anything, MPT70 induced higher responses than ESAT-6 (Fig. 3a). However, 3 weeks after Mtb Erdman challenge, there was a bigger proportion of ESAT-6-specific T cells in the lung compared to MPT70 T cells, indicating earlier expansion/recruitment of ESAT-6-specific T cells (although this was not statistically significant) (Fig. 3b). A characterization of the MPT70- and ESAT-6-specific CD4 T cells showed that there was no difference in T cell differentiation preinfection (Fig. 3c), indicating that there was no intrinsic antigen effect on this parameter. In contrast, a similar analysis postinfection revealed that vaccination with ESAT-6 had a greater impact on lowering the antigen-specific T cell differentiation in this setting (Fig. 3d). Similar to the observation in Fig. 2a, MPT70-specific T cells had an FDS of around 2.7 during early infection, and vaccination did not change this noticeably. In unvaccinated animals, this level was sustained at week 20, while vaccination with MPT70 lowered this to 1.1. In contrast, the FDS of ESAT-6-specific T cells remained high throughout infection (6.9 at week 3 and 5.8 at week 20), while vaccination with ESAT-6 resulted in a substantially lower differentiation level around 0.8 to 1.8 throughout the infection (Fig. 3d). These observations were confirmed by KLRG1 staining, which showed a similar pattern to FDS (Fig. S4a). Finally, we determined the protective efficacy by plating lung homogenates. Of note, at weeks 3 to 4, we observed significant protection of both ESAT-6 (*P* < 0.0001) and MPT70 (*P* = 0.0001), demonstrating the vaccine potential of both antigens (Fig. 3e). However, over the course of four independent experiments, bacterial burdens were lower in ESAT-6-vaccinated animals than MPT70-vaccinated animals (*P* = 0.047 at 3 to 4 weeks postinfection [p.i.]) (Fig. 3e and Fig. S4b).

10.1128/mBio.00226-21.4 FIG S4 Long-term vaccine impact of ESAT-6 and MPT70 during Mtb infection. CB6F1 mice were vaccinated with MPT70, ESAT-6, or saline three times and challenged with Mtb Erdman 6 weeks after the third immunization. (a) Percentage of KLRG1^+^ PD-1^−^ of MPT70- or ESAT-6-specific CD4 T cells in vaccinated mice and mice treated with saline 3 and 20 weeks after Mtb infection (*n* = 4). Box plots with whiskers indicating the minimum and maximum values are shown. (b) The bacterial burdens were determined in the lungs of mice treated with saline and vaccinated mice 19 or 20 weeks after Mtb infection (*n* = 28). The graph shows the results from four individual experiments. One-way ANOVA with Tukey’s multiple-comparison test was used to assess significant differences. Download FIG S4, TIF file, 0.2 MB. Copyright © 2021 Strand Clemmensen et al. 2021 Strand Clemmensen et al. https://creativecommons.org/licenses/by/4.0/ This content is distributed under the terms of the Creative Commons Attribution 4.0 International license.

Taken together, vaccination with ESAT-6 had the highest impact on T cell differentiation during subsequent infection, and while vaccination with both antigens induced robust protection, there was better protection with ESAT-6. Although observed with antigens of different size and immunogenicity, this suggests that *in vivo* antigen expression could regulate T cell quality as well as protective capacity.

### Constitutive expression of MPT70 accelerates T cell differentiation and improve vaccine protection.

**FIG 4:**
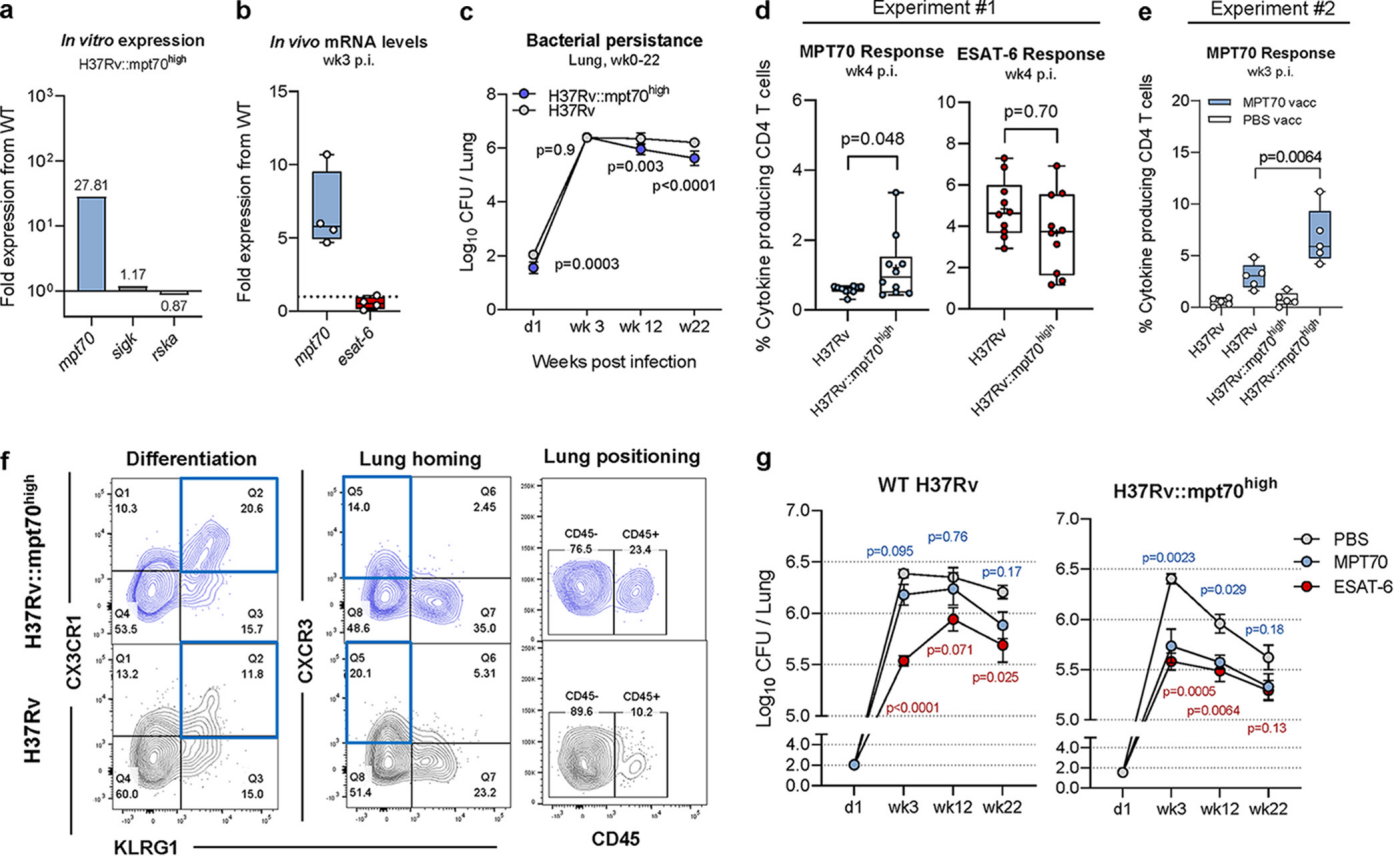
Overexpression of MPT70 accelerates T cell differentiation and increases bacterial susceptibility to MPT70 vaccination. (a) *In vitro* fold gene expression of *sigK*, *rskA*, and MPT70 in H37Rv::mpt70^high^ compared to WT H37Rv. All genes were tested in technical duplicates and normalized to *esxA* expression using primers in Table 1. (b) *In vivo* fold gene expression of MPT70 and ESAT-6 in the lungs of H37Rv::mpt70^high^-infected mice compared to WT H37Rv-infected mice 3 weeks after Mtb challenge (*n* = 4). Genes were analyzed in technical duplicates using primers and probes in Table 2, normalized to 16S rRNA expression, and shown as fold increase from WT H37Rv. A two-tailed, unpaired *t* test was used to assess statistical differences. (c) Bacterial burden in the lungs of mice injected with PBS at day 1, week 3, week 12, and week 22 after infection with either H37Rv:: mpt70^high^ or WT H37Rv infection (*n* = 5). Values shown are means ± SEM. Multiple *t* tests with correction for multiple testing using the Holm-Sidak method were used to assess statistical differences. (d) Frequency of lung MPT70- and ESAT-6-specific CD4 T cells 4 weeks after Mtb infection (*n* = 10). Box plots with whiskers indicating the minimum and maximum values are shown. The mean values are indicated with + symbols. A unpaired, two-tailed *t* test was used to assess statistical differences. (e) Frequency of lung MPT70-specific CD4 T cells 3 weeks after Mtb infection in mice injected with PBS (white boxes) and mice vaccinated with MPT70 (blue boxes) (*n* = 5). A unpaired, two-tailed *t* test was used to assess statistical differences. (f) Representative concatenated FACS plots (*n* = 10) showing the expression of CX3CR1, CXCR3, KLRG1, or CD45 on MPT70-specific CD4 T cells 4 weeks after H37Rv:: mpt70^high^ infection (blue) or H37Rv infection (gray). Flow cytometry gating is shown as depicted in Fig. S1a, using antibodies shown in Table 5. (g) Bacterial numbers were determined in the lungs of mice vaccinated with PBS or mice vaccinated with MPT70 and ESAT-6 at day 1, week 3, week 12, and week 22 after WT H37Rv infection (left) or H37Rv::mpt70^high^ infection (right) (*n* = 4 to 5). One mouse was excluded from the week 12 time point (H37Rv::mpt70^high^, MPT70 vaccinated), as the mouse was very sick, had high weight loss, and met the study’s predefined humane endpoints (*P* value = 0.67, if included). Values shown are means ± SEM. Statistical differences were assessed using one-way ANOVA with Tukey’s multiple-comparison test.

To investigate whether T cell differentiation and vaccine protection is directly linked to *in vivo* antigen transcription, we utilized a recently engineered H37Rv strain (36) (herein called H37Rv::mpt70^high^) with significantly increased *in vitro* expression of MPT70 due to insertion of *sigK* (Rv0445c) and *rskA* (Rv0444c) from Mycobacterium orygis (6, 36). In line with the previous report (36), we observed that this strain upregulated *in vitro* expression of MPT70 compared to wild-type (WT) H37Rv, while very little change was observed for the regulators of MPT70 transcription (SigK and RskA) (Fig. 4a). From this, we anticipated that the H37Rv::mpt70^high^ strain would have an increased early *in vivo* expression of MPT70. Transcription analysis of mRNA from lungs of mice 3 weeks after aerosol infection confirmed this, as MPT70 was 6.7-fold higher expressed by mice infected with H37Rv::mpt70^high^ than in mice infected with WT H37Rv (Fig. 4b). This analysis also confirmed the observations with Mtb Erdman in Fig. 1a, showing that expression of MPT70 in WT H37Rv was very low at week 3 in contrast to ESAT-6 (Fig. S5a). Of note, complementation of H37Rv did seem to affect the bacterial fitness *in vitro* (Fig. S5b), which was also associated with a small, but detectable, difference in CFU at day 1 (Fig. 4c). Interestingly, H37Rv::mpt70^high^ and WT H37Rv had similar *in vivo* growth up until week 3, but overexpression of MPT70 seemed to impact long-term persistence negatively (Fig. 4c). We next analyzed the impact on T cell responses in two independent experiments. Since bacterial load is expected to influence T cell differentiation (21–23), we focused our analysis around week 3 postinfection, where the number of bacteria was the same for WT H37Rv and H37Rv::mpt70^high^. The first experiment demonstrated that the CD4 T cell response against MPT70 was significantly increased in mice infected with H37Rv::mpt70^high^ compared to WT H37Rv (Fig. 4d). For comparison, ESAT-6 responses did not differ between the two strains (Fig. 4d). In agreement with this observation, a second experiment showed that the response to MPT70 vaccination was increased in mice infected with H37Rv::mpt70^high^ (*P* = 0.0064) compared to WT-infected mice (Fig. 4e). We then asked whether the early expression and elevated MPT70 immune response accelerated CD4 T cell differentiation and altered expression of markers associated with lung homing. CXCR3 is primarily expressed on lung-homing T cells (25, 26), whereas CX3CR1 is associated with T cells in the vasculature (37). Studying these surface markers on MPT70-specific CD4 T cells revealed a substantially higher proportion of CX3CR1^+^KLRG1^+^ T cells after H37Rv::mpt70^high^ infection (20.6%) compared to H37Rv infection (11.8%), indicating increased differentiation and decreased lung homing capacity for H37Rv::mpt70^high^-primed MPT70 CD4 T cells (Fig. 4f). This was also evident in a vaccination setting, as MPT70 immunization induced the biggest reduction of CX3CR1^+^KLRG1^+^ T cells after H37Rv::mpt70^high^ infection (Fig. S5c). In contrast, the frequency of CX3CR1^+^KLRG1^+^ ESAT-6 T cells was not different between the strains, demonstrating that the increased T cell differentiation was a specific effect of MPT70 overexpression (Fig. S5d). In line with increased MPT70-specific T cell differentiation, a lower frequency of CXCR3-expressing CD4 T cells was found in H37Rv::mpt70^high^-infected animals (14.0%) compared to H37Rv-infected animals (20.1%), which also correlated with a higher proportion of CD45-labeled MPT70 CD4 T cells located in the lung-associated vasculature (23.4% to 10.2%) (Fig. 4f). Together, the immune data showed that increased early *in vivo* expression altered the MPT70-specific immune responses to resemble those of ESAT-6 after WT Mtb infection. We finally addressed whether vaccine-induced protection of MPT70 was increased if mice were challenged with the H37Rv::mpt70^high^ strain. Immunized mice were challenged with either WT H37Rv or H37Rv::mpt70^high^, and bacterial numbers were determined in the lungs at weeks 3, 12, and 22. Consistent with data from Mtb Erdman (Fig. 3e), MPT70 vaccination conferred less protection than ESAT-6 against challenge with WT H37Rv (Fig. 4g). In contrast, MPT70 vaccination induced a substantial reduction in bacterial load at all time points in mice challenged with H37Rv::mpt70^high^, providing protection that was comparable to protection by ESAT-6.

10.1128/mBio.00226-21.5 FIG S5 Characterization of the modified H37Rv::mpt70^high^ strain. (a) Relative mRNA levels of MPT70 and ESAT-6 in the lungs of WT H37Rv- and H37Rv::mpt70^high^-infected mice 3 weeks after aerosol Mtb challenge (*n* = 5). mRNA levels were normalized to 16S rRNA. Box plots with whiskers indicating the minimum and maximum values are shown. A paired, two-tailed *t* test was used to assess significant differences. (b) *In vitro* growth of WT H37RV, H37Rv::mpt70^high^ (*rskA* and *sigK* insert of *M. orygis* origin), and H37Rv::Rv (*rskA* and *sigK* insert of Mtb origin). Strains were grown in 7H9 medium for 4 days, and the OD_600_ was measured every 24 h (*n* = 3). Values shown are means ± standard deviations (SD). Multiple *t* tests with correction for multiple tests using the Holm-Sidak method were used to assess significant differences. (c) KLRG1^+^ CX3CR1^+^ expressing MPT70-specific CD4 T cells in PBS-treated and MPT70-vaccinated mice 3 weeks after WT H37RV and H37Rv::mpt70^high^ infection (*n* = 5). The values for individual mice (symbols) and the average mean are shown. (d) KLRG1^+^ CX3CR1^+^ expressing MPT70- and ESAT-6-specific CD4 T cells in mice injected with PBS 3 to 4 weeks after WT H37RV and H37Rv::mpt70^high^ infection (*n* = 5 to 10). Box plots with whiskers indicating the minimum and maximum values are shown. Two independent experiments were performed. An unpaired, two-tailed *t* test was used to assess significant differences. Download FIG S5, TIF file, 0.3 MB. Copyright © 2021 Strand Clemmensen et al. 2021 Strand Clemmensen et al. https://creativecommons.org/licenses/by/4.0/ This content is distributed under the terms of the Creative Commons Attribution 4.0 International license.

Taken together, overexpression of MPT70 increased MPT70 vaccine protection substantially, indicating that *in vivo* antigen expression kinetics regulates vaccine protection rather than properties of the antigen as such. This, even though high antigen expression resulted in increased T cell differentiation. Vaccination with highly expressed antigens therefore requires that the vaccine primes T cells of a low differentiation.

## DISCUSSION

CD4 T cell differentiation is a key determinant of protective immunity against Mtb (21, 38, 39), and antigen load is described to influence T cell development (21–23). Importantly, during the course of infection, Mtb adapts to the changing environment of the host, and it is poorly described how differential *in vivo* antigen expression influences CD4 T cell responses and vaccine potential. In this study, we compared ESAT-6, which is a well-known constitutively expressed antigen (32, 33), to MPT70 that is expressed at negligible amounts *in vitro* but inducible upon IFN-γ activation (5, 6) or nutrient deprivation (8) in infected macrophages. After murine aerosol infection, we observed that MPT70 immune recognition was delayed compared to ESAT-6, which correlated with lower initial antigen expression of MPT70. This is in line with a previous genome-wide microarray study after H37Rv infection in BALB/c mice, where MPT70 expression gradually increases from day 7 to 28, where it is similar to ESAT-6 (40). Based on this, it can be speculated that *in vivo* expression of MPT70 is upregulated in response to host adaptive immune responses, which occur around weeks 2 to 3 in mice (41, 42). This likely places MPT70 in the same functional category as the stress-induced genes encoded by the dormancy survival regulon (DosR) that likewise are upregulated in chronically infected mice and IFN-γ-treated macrophages (5, 43).

Given that antigen availability influences T cell quality, we asked how the delayed antigen recognition of MPT70 would impact the CD4 T cell response. In mice infected with WT Mtb Erdman, we observed that MPT70-specific CD4 T cells had a significantly lower differentiation status than ESAT-6 based on the expression of KLRG1, CXCR3, CX3CR1, and T-bet as well as cytokine expression pattern. This is in line with our most recent results, showing that MPT70 is highly immunogenic during late chronic infection, but with an altered T cell phenotype compared to ESAT-6 (29). Having established associations between antigen expression and T cell quality, we investigated whether there was a causal relationship. For this, we utilized a newly described H37Rv strain, which has high *in vitro* expression of MPT70 due to a gene insert of the regulators *sigK* (Rv0445c) and *rskA* (Rv0444c) from M. orygis (36). Importantly, we observed that infection with this strain significantly increased the differentiation state of the MPT70-specific CD4 T cells and diminished their ability to enter the infected lung tissue. No differences were observed for ESAT-6-specific CD4 T cells, demonstrating that this was a specific consequence of overexpressing MPT70. These data therefore indicate that the quality of infection-driven T cells is dictated by the *in vivo* antigen expression profile. This is in line with the study by A. Moguche et al., demonstrating that *in vivo* overexpression of Ag85B significantly increased CD4 T cell differentiation (22). Given that Ag85B expression is downregulated early during infection (32), in contrast to MPT70, these studies collectively suggest that CD4 T cell differentiation is a result of the cumulative antigen exposure, and that highly and constitutively expressed antigens would have the highest degree of T cell differentiation.

We finally examined the link between antigen expression and protective capacity in a vaccination setting. Antigens expressed during late-stage Mtb infection have been the focus of multistage TB vaccines as they may specifically target bacteria during latency (16, 44–46), and the expression profile of MPT70 could make this an interesting candidate. After vaccination, both MPT70 and ESAT-6 induced robust protection against Mtb Erdman infection, which is in line with other studies reporting high vaccine potential of these two antigens (13, 15, 29, 30). However, despite lower immunogenicity, ESAT-6 vaccination induced the highest protection. Although ESAT-6 and MPT70 are different in both size, epitope pattern, and immunogenicity, this observation prompted us to hypothesize that constitutive *in vivo* antigen expression is optimal for vaccine protection. To investigate this more directly, we compared the effect of immunization with MPT70 against WT H37Rv or H37Rv::mpt70^high^ where MPT70 is overexpressed. Here we observed that the MPT70-mediated protection was significantly increased against the H37Rv::mpt70^high^ strain, where ESAT-6 and MPT70 performed similarly, compared to the WT H37Rv strain. Together with the T cell analysis, these data suggest that constitutively expressed antigens are superior vaccine antigens, supposedly because of increased antigen “visibility” by the infected macrophages, but due to continuously high antigen presence, the infection-driven T cells are also pushed toward terminal differentiation and decreased functionality. We speculate that this could be a survival strategy by Mtb (47) and show how targeted vaccination with adjuvanted proteins can compensate for this by priming (and maintaining) less differentiated T cells (29). Although the mechanisms behind this are unknown, previous data confirm that immunization with Mtb proteins in the CAF01 adjuvant induces CD4 T cells of low differentiation as well as Th17/Th17-like T cells that are maintained during Mtb infection (41, 48, 49). Although this somewhat contrasts with data for memory CD8 T cells (50), CD4 T cell plasticity is maximal at early stages of differentiation, and it has been shown that Th17 cells and less differentiated Th17-like T cells share many features with stem cells and have the capacity to persist over longer periods of time (48, 51–53). Together, these features could potentially explain the observed ability of CAF01-induced CD4 T cells to resist infection-driven T cell differentiation, which will be an important research topic of the future.

Importantly, we investigated only the impact of vaccination after single antigen immunization, and it can be speculated that MPT70, and similar antigens, might perform differently in larger fusions proteins. Here, the accelerated adaptive immune responses offered by other antigens, like ESAT-6 (41), may trigger earlier expression of MPT70, which in turn would increase the MPT70-mediated protection against Mtb. Additionally, MPT70 is naturally overexpressed in the animal-adapted *Mycobacterium* strains; *M. orygis*, M. caprae, and M. bovis (6), which could imply that an MPT70-containing vaccine would be particularly efficacious against pathogens causing TB in livestock.

The course of chronic infection mimicked in the mouse model is in many ways different from the human Mtb infection that can last for years and display distinct features in granuloma structure and environment (54). In fact, in nonhuman primates (NHPs), it has been reported that very few of the Mtb-specific CD4 T cells are terminally differentiated, and in contrast to mice, almost all of the Mtb-specific cells are found in the lung parenchyma (55). This could lead to the interpretation that T cell differentiation is of less importance in NHPs compared to mice. However, it has been shown that a vaccine induction of less differentiated central memory T cells is associated with increased protection in NHPs (56), and so far, it has not been studied whether terminal T cell differentiation occur in NHPs after long-term infection (i.e., after day 49). Indeed, in humans, the degree of CD4 T cell differentiation is increased in people with active disease compared to latent tuberculosis infection (57–59), indicating that progression of infection drives T cell differentiation. Furthermore, increased Mtb-specific CD4 T cell differentiation has been associated with increased tissue pathology in TB patients (60). Together, these studies support that T cell differentiation is an important feature of the protective response in humans, even if defective T cell homing is not part of the explanation. This warrants further validation of our results in higher animal models and/or humans.

Besides host species, there may also be differences in antigen expression levels between clinical Mtb isolates that are known to display some level of genetic diversity and virulence variability (61), and future studies should extrapolate our results to other relevant isolates. Of note, MPT70 has been described as part of the “core transcriptome” in macrophage phagosomes with conserved expression and regulation across all MTBC isolates (62), suggesting that *in vivo* MPT70 expression will not vary between clinical Mtb isolates. Overexpression of MPT70, however, did seem to impact the bacterium’s overall capability to persist after week 3, implicating that abundant MPT70 is not advantageous for Mtb during chronic infection. This has also been seen in a similar study overexpressing Mtb heat shock proteins (63) and might be CD4 T cell dependent as has been reported for an Ag85B-overexpressing strain (64).

Overall, our study provides new insights into host-pathogen interactions and describes how *in vivo* antigen expression kinetics can regulate T cell functionality and vaccine protection. Data show that high antigen expression drives T cells toward terminal differentiation and that targeted preventive vaccination can counteract this effect. We also demonstrate that highly expressed antigens are optimal vaccine targets and accentuate that T cell differentiation can be used as a new way to identify the most promising antigens. This has implications for rational vaccine design, and future preclinical and clinical studies could use antigen-specific T cell differentiation as a readily measurable proxy for high *in vivo* antigen expression.

## MATERIALS AND METHODS

### Mice.

Six- to 8-week-old female CB6F1 mice (BALB/c × C57BL/6; Envigo) or C57BL/6 mice (Jackson Laboratory) were purchased. Mice were randomly assigned to cages of five to eight on the day of arrival. Before initiating the experiment, mice had at least 1 week of acclimation. Mice were housed in biosafety level 2 (BSL II) in individually ventilated cages (Scanbur, Denmark) and had access to nesting material as well as enrichment. During the course of the experiment, mice were fed with irradiated Teklad global 16% protein rodent diet (Envigo, catalog no. 2916C) and had access to water *ad libitum*. On the day of challenge, cages with mice were transferred to BSL-III where they were housed until termination of the experiment.

### Ethics.

All experimental protocols were initially reviewed and approved by a local ethical committee at Statens Serum Institut (SSI) and by the Facility Animal Care Committee at the Research Institute of McGill University Health Center (RI-MUHC) (project identifier [ID] 2015-7656). Experimental procedures were conducted in accordance with the regulations set forward by the Danish Ministry of Justice, Canadian Council of Animal Care (CCAC), and Animal Protection Committees under license permit no. 2019-15-0201-00309 and in compliance with the European Union Directive 2010/63/EU.

### Recombinant proteins and immunizations.

The following recombinant antigens were expressed and purified as previously described (29): ESAT-6 (Rv3875) or MPT70 (Rv2875). Mice were immunized three times subcutaneously (s.c.) at the base of the tail or neck at 2-week intervals with either recombinant ESAT-6 or MPT70. Recombinant proteins were diluted in Tris-HCl buffer plus 9% trehalose (pH 7.2) and adjuvanted with cationic adjuvant formulation 1 (CAF01) consisting of dimethyldioctadecylammonium (DDA) and trehalose dibehenate (TDB) at a ratio of 250 μg DDA per/50 μg TDB (35). As a control, mice were vaccinated with phosphate-buffered saline (PBS).

### Mtb infections and CFU enumeration.

Mtb Erdman (ATCC 35801/TMC107) was cultured in Difco Middlebrook 7H9 (Becton Dickinson [BD]) supplemented with 10% BBL Middlebrook ADC Enrichment (BD) for 2 to 3 weeks using an orbital shaker (∼110 rpm, 37°C). Bacteria were harvested in log phase and stored at −80°C until use. Before use in the experiment, the concentration of the bacterial stock was determined by plating in triplicate. For aerosol infections, the vial of Mtb was thawed, sonicated for 5 min, thoroughly suspended with a 27-gauge (27G) needle to remove clumps, and mixed in PBS to the desired concentration. Mice were challenged with 0.5 × 10^6^ CFU/ml (around 50 to 100 CFU) Mtb Erdman by the aerosol route using a Biaera exposure system controlled via AeroMP software.

The Mtb Pasteur H37Rv, Pasteur H37Rv::mpt70^high^ (*rskA* and *sigK* of *M. orygis*) (36), and Pasteur H37Rv::Rv (*rskA* and *sigK* of M. tuberculosis) (36) strains were grown in Middlebrook 7H9 medium (Difco Laboratories) supplemented with 0.05% Tween 80 (Sigma-Aldrich), 0.2% glycerol, 10% ADC Enrichment (BD) in a rolling incubator at 37°C. The bacterial cultures were passaged twice, adjusted to an optical density at 600 nm (OD_600_) of 0.5, pelleted, resuspended in glycerol, and subsequently frozen at −80°C. On the day of the experiment, vials were thawed, thoroughly resuspended with a 27G needle, and adjusted to an OD of 0.05 in PBS (approximately 50 CFU). Mice were challenged with an aerosol infection using a CH Technologies nose-only inhalation exposure system with 15-min exposure, up to 18 mice per run. Mice euthanized for each experimental time point were in the same aerosol run.

To enumerate bacteria in the lungs of mice after infection, left lobes from individual mice were homogenized with GentleMACS M-tubes (Miltenyi Biotec) in 3 ml MilliQ water containing PANTA antibiotic mixture (BD, catalog no. 245114) or with an Omni Tissue Homogeniser and Hard Tissue Omni Tip Plastic Homogenising Probes (Omni International) in a 50-ml tube containing 1 ml 7H9 supplemented medium. The homogenate was serially diluted, plated, and grown on 7H11 plates (BD) or 7H10 plates containing PANTA for approximately 2 to 3 weeks at 37°C and 5% CO_2_. CFU were counted, log transformed to normalize data, and shown as log_10_ CFU per the whole lung. Whenever possible, a cutoff of 10 colonies was set to minimize variability and errors due to plating.

### *In vitro* growth assay.

The growth of Mtb H37Rv, Mtb H37Rv::mpt70^high^, and H37Rv::Rv in 7H9 medium (supplemented as described above) was monitored with a spectrophotometer at OD_600_ in triplicates every 24 h for 4 days. The culture flasks were incubated at 37°C under rotating conditions.

### *In vitro* and *in vivo* Mtb RNA extractions.

For *in vitro* RNA extractions, Mtb H37Rv and H37Rv::mpt70^high^ were passaged twice and adjusted to an OD_600_ of 0.2 to 0.5. The bacteria were pelleted and resuspended in 1 ml TRIzol reagent (Invitrogen, catalog no. 15596026). For *in vivo* RNA extractions, the postcaval lung lobes of Mtb-infected mice were harvested aseptically and immediately stored at −80°C in 1 ml RNA later (Qiagen, catalog no./ID: 76106) until further processing. The lung tissues were mechanically disrupted in 1 ml TRIzol reagent using lysing matrix D or E tubes (MP Biomedicals, stock-keeping unit [SKU] 116913050-CF, SKU 116914050-CF) and a FastPrep-24 bead beater (MP, SKU 116004500). The grinded lung tissues were stored at −80°C until further processing. On the day of RNA extraction, the lung tissues were bead beated with 0.1-mm zirconia/silica beads three times at 6.5 m/s for 30 s with 3-min rest on ice in between runs. The beads were pelleted by centrifuging at 12,000 × *g* for 1 min, and the TRIzol layer was moved to a fresh tube containing chloroform isoamyl alcohol (24:1). After centrifuging at 12,000 × *g* for 15 min at 4°C, the top aqueous phase was transferred and precipitated with 3 M sodium acetate and isopropanol for at least 2 h or overnight at –20°C. The pellet was washed twice in ethanol, air dried, and resuspended in RNase-free water. A cleanup step was performed with RNA Easy minikit (Qiagen), followed by a minimum of three DNase treatments (Ambion, catalog no. AM1907). The RNA purity and concentration were measured by spectrophotometry (Tecan Infinite M200 Pro plate reader) or using the RNA Qubit assay (Invitrogen, catalog no. Q32852). RNA samples were checked for remaining genomic DNA by PCR using the SigA primers. A total of 300 to 1,000 ng RNA was reverse transcribed (Thermo Scientific, catalog no. K1621); a minus reverse transcriptase control was included for every sample.

### Real-time qPCR and gene expression analysis.

To determine *in vitro* mRNA levels of MPT70, SigK, RskA, and ESAT-6, we performed a reverse transcription-quantitative PCR (RT-qPCR) using Maxima SYBR green kit (Thermo Scientific, catalog no. K0223) with the use of the following primers (Table 1). mRNA levels were normalized to EsxA and fold gene expression from Mtb H37Rv was plotted as 2^−ΔCt^.

**TABLE 1:** Primers used for *in vitro* gene expression analyses

Gene	Primer sequence	
	Forward primer (5′–3′)	Reverse primer (5′–3′)
*sigK*	GGTGGGCCATGGTCAAAA	AGTTTGACTCCGCCAAAGGTT
*rskA*	ACACAGGTCTGCTGGTGATG	TTCGACGGTGAATGCCAGTG
*mpt70*	CCTCGAACAATCCGGAGTTG	GTAGACACCCCACCACAGAC
*esat-6*	CAGAGCAGCAGTGGAATTTCG	CATTTTTGCTGGACACCCTGG
*sigA*	ATCGCGCGAAAAACCATCTG	CACCGACTGCAGTTGATCCT

*In vivo* mRNA levels were measured with qRT-PCR using dually labeled probes (Eurofins) (Table 2). All probes and primers were diluted to a final concentration of 250 nM (probes) and 900 nM (primers), respectively, and mixed with either iTaq Universal Probe Supermix (Bio-Rad, catalog no. 1725130) or SsoAdvanced Universal Probes Supermix (Bio-Rad, catalog no. 1725281). All cDNA samples were prediluted 10 times in diethyl pyrocarbonate (DEPC)-treated water and used in a final dilution of 1:40 in the reaction. Thermal cycling protocol was programmed according to the manufacturer’s instructions for low abundant targets (45 cycles with 1 cycle consisting of 30 s at 95°C, 10 s at 95°C, and 1 min at 60°C). For gene expression analysis throughout Mtb Erdman infection (Fig. 1a), average quantification cycle (*C_q_*) values for each sample were normalized to 16S rRNA and shown as relative mRNA levels (2^−ΔCq^). The fold gene expression of *mpt70* and *esat-6* in H37Rv::mpt70^high^ from WT H37Rv were calculated with the 2^−ΔΔ^*^CT^* method with normalization to 16S rRNA (Fig. 4b). For every run, a no-template control, negative control (naive mouse), and positive controls (genomic DNA of H37Rv and BCG) were included.

**TABLE 2:** Primers and probes used for *in vivo* gene expression analyses

Target	Primer or probe sequence			Reference
	Forward primer 5′–3′	Reverse primer 5′–3′	FAM-BHQ1 probe	
16s rRNA	TCCCGGGCCTTGTACACA	CCACTGGCTTCGGGTGTTA	CGCCCGTCACGTCATGAAAGTCG	66
*esat-6*	ATGACAGAGCAGCAGTGG	CGTCAAGGAGGGAATGAATG	AGCGCAATCCAGGGAAATGTCACGTC	
*mpt70*	GCTCAATCCGCAAGTAAACC	CCGGCAGCTTGCTAAATG	CCTCAACAGCGGTCAGTACACGGTGTTC	

### *In vivo* labeling of intravascular CD4 T cells.

Mice were anesthetized with isoflurane and injected intravenously with 2.5 to 5.0 μg fluorescein isothiocyanate (FITC)-labeled CD45 antibody (BD Pharmingen, clone 104; catalog no. 553772) diluted in 100 to 250 μl PBS. Three minutes after injection, mice were euthanized by cervical dislocation, and organs were aseptically harvested for further processing as described below.

### Preparation of single-cell suspensions.

Spleens or lungs were aseptically harvested from euthanized mice. Lungs were first homogenized in Gentle MACS tubes C (Miltenyi Biotec) or chopped into small pieces using scalpels, followed by 1 h of collagenase digestion (Sigma-Aldrich; catalog no. C5138) at 37°C and 5% CO_2_. The lung homogenate and spleens were forced through 70- to 100-μm cell strainers (BD Biosciences) with the stopper from a 5-ml syringe (BD) and washed twice with cold RPMI medium (Gibco; RPMI 1640) by centrifuging 5 min at 1,800 rpm. A red blood cell lysis step was performed in between washes (Roche, catalog no. 11814389001). Cells were finally resuspended in enriched RPMI medium (RPMI 1640, 10% heat-inactivated fetal calf serum (FCS) (Biochrom GmbH), 10 mM HEPES (Invitrogen), 2 mM l-glutamine (Invitrogen), 1 mM Natriumpyruvate (Invitrogen), 1× nonessential amino acids (MP Biomedicals, LLC), 5 × 10^5^ M 2-mercaptoethanol (Sigma-Aldrich), and penicillin-streptomycin (Gibco). Cells were counted using an automatic Nucleocounter (Chemotec) and adjusted to 2 × 10^5^ cells/well for enzyme-linked immunosorbent assay (ELISA) and 1  × 10^6^ to 2 × 10^6^ cells/well for flow cytometry.

### Design of an MHC-II tetramer specific for MPT70.

Splenocytes of MPT70-vaccinated mice were restimulated in the presence of overlapping 15-mer peptides, and two murine epitopes were identified (29). The recognized peptides corresponded to amino acid (aa) positions 37 to 53 and 93 to 109 in MPT70. These epitopes were further epitope mapped in vaccinated mice with peptides varying 1 aa in length to identify the minimal core epitope which is the minimal number of amino acids necessary for T cell recognition (see Fig. S2a in the supplemental material). We found EYAAANPTGPA and FAPTNAAF as the core epitopes, but as FAPTNAAF was not highly recognized by Mtb-infected mice (data not shown), it was not further characterized. As the optimal peptide length for an MHC-II tetramer may vary between 11 and 16 aa, different peptide lengths extending the core epitope were tested with no big difference in response magnitude as long as the sequence “YAAANPTGP” was present (Fig. S2b). On the basis of these results, we designed a tetramer specific for I-A^b^:MPT70_38-52_ (CAEYAAANPTGPAS). This epitope has previously been identified in MPB70 DNA-immunized C57BL/6 H-2^b^ and B6D2 (F1) H-2d^b^ mice in an enzyme-linked immunosorbent spot (ELISPOT) assay with no humoral response detected in mice of haplotype H-2^d^ (65).

### MHC-II tetramer staining.

Tetramers (MPT70^38-52^:I-Ab, ESAT-6^4-17^:I-Ab) conjugated to BV421 or phycoerythrin (PE) and corresponding negative controls (hCLIP:I-Ab) were provided by the NIH Tetramer Core Facility (Atlanta, GA, USA). MHC-II tetramers were titrated and tested for optimal staining conditions before the experiment. Single-cell suspensions were stained with tetramers diluted 1:50 in fluorescence-activated cell sorting (FACS) buffer (PBS plus 1% FCS) containing 1:200 Fc block (anti-CD16/CD32) for 30 min at 37°C. The MPT70^38-52^ MHC-II tetramer was specifically developed for this study (Fig. S2).

### *In vitro* restimulation and intracellular cytokine staining.

For intracellular cytokine staining (ICS), cells were restimulated with 2 μg/ml antigen or medium in the presence of 1 μg/ml anti-CD28 (clone 37.51) and anti-CD49d (clone 9C10-MFR4.B) in 96-well V-bottom TCT microtiter plates (Corning; catalog no. 3894) for 1 h at 37°C and 5% CO_2_. Restimulation with ionomycin in conjunction with phorbol myristate acetate (PMA) was included as a positive control. Subsequently, 10 μg/ml Brefeldin A was added to each well (Sigma-Aldrich; catalog no. B7651) (5 mg) and followed by another 5- to 6-h incubation at 37°C and 5% CO_2_, after which cells were kept at 4°C until staining or immediately surface stained and fixed.

Prior to staining, cells were washed with FACS buffer and subsequently stained surface markers diluted in brilliant stain buffer (BD Horizon; catalog no. 566349) using antibodies indicated in Tables 3, 4, and 5. Fixation and permeabilization were performed using the Fixation/Permeabilization Solution kit (BD Cytofix/Cytoperm; catalog no. 554714) or Foxp3/Transcription Factor Staining Buffer Set (eBioscience; catalog no. 00-5523-00) per the manufacturer’s instructions followed by intracellular staining with anti-IFN-γ, anti-IL-2, anti-TNF-α, and anti-IL17A and/or transcription factor staining with anti-T-bet. Fluorescence minus one controls were performed for CD3, CD44, KLRG1, PD-1, CXCR3, CX3CR1, IL-2, IL-17, IFN-γ, and T-bet on pooled cells to set boundary gates for surface, intracellular, and transcription factor markers. Gating strategies for defining tetramer-positive CD4 T cells and cytokine-producing CD4 T cells are exemplified in Fig. S3 (tetramer) and Fig. S1 (ICS).

**TABLE 3:** Antibodies used for tetramer characterization in Fig. 2

Antibody	Fluorophore	Supplier	Clone	Dilution	Catalog no.
CD3	Bv650	Biolegend	17A2	1:100	100229
CD4	Bv510	Biolegend	RM4-5	1:400	100559
Viability	efluor780	eBioscience		1:1000	
CXCR3	PerCpCy5.5	eBioscience	CXCR3-173	1:100	45-1831-82
CX3CR1	Bv785	Biolegend	SAO11F11	1:100	149029
CD44	Alx700	Biolegend	IM7	1:200	103026
KLRG1	Bv711	Biolegend	2F1	1:100	138427
CD45 (*in vivo*)	FITC	BD Bioscience	104	1:50	553772
T-bet	PE-Cy7	eBioscience	4B10	1:100	25-5825-82
hClip Control	Bv421	NIH		1:50	
MPT70 tet	Bv421	NIH		1:50	
hClip Control	PE	NIH		1:50	
E6 tet	PE	NIH		1:50	

**TABLE 4:** Antibodies used for T cell characterization in Fig. 1, 2, and 3

Antibody	Fluorophore	Supplier	Clone	Dilution	Catalog no.
CD3	Bv650	Biolegend	17A2	1:100	100229
CD4	Bv510	Biolegend	RM4-5	1:400	100559
CD44	Alx700	Biolegend	IM7	1:200	103026
KLRG1	Bv711	Biolegend	2F1	1:100	138427
PD-1	Bv421	Biolegend	29F.1A12	1:100	135218
IL-2	APC-Cy7	BD Bioscience	JES6-5H4	1:100	560547
IL-17	PerCpCy5.5	eBioscience	XMG1.2	1:200	45-7177-82
TNF-α	PE	eBioscience	MP6-XT22	1:200	12-7321-82
IFN-y	PE-Cy7	eBioscience	XMG1.2	1:200	25-7311-82
CD45.2	FITC	BD Bioscience	104	1:50	553772

**TABLE 5:** Antibodies used for T cell characterization in Fig. 4.

Antibody	Fluorophore	Supplier	Clone	Dilution	Catalog no.
Viability	440UV	BD Horizon		1:1,000	566332
CD8a	APC-H7	BD Pharmingen	53-6.7	1:100	560182
CD4	PE	BD Pharmingen	GK1.5	1:400	557308
CD45	FITC	BD Pharmingen	30-F11	1:10	553080
CD44	BUV395	BD OptiBuild	IM7	1:100	740215
KLRG1	BB700	BD OptiBuild	2F1	1:100	742199
PD-1 (CD279)	APC-R700	BD Horizon	J43	1:100	565815
CXCR3 (CD183)	BUV737	BD OptiBuild	CXCR3-173	1:100	741895
CX3CR1	PE/Dazzle 594	Biolegend	SA011F11	1:100	149014
CD3e	V500	BD Horizon	500A2	1:50	560771
TNF-α	PE-Cy7	BD Pharmingen	MP6-XT22	1:200	557644
IFN-y	Bv421	BD Horizon	XMG1.2	1:100	563376
IL-17A	BV786	BD Horizon	TC11-18H10	1:100	564171
IL-2	APC	eBioscience	JES6-5H4	1:100	17-7021-82

### IFN-γ sandwich ELISA.

Splenocytes or lung cells were adjusted to a cell concentration of 2 × 10^5^ cells/well and restimulated in the presence of recombinant protein or peptides in round-bottom plates for 3 days as previously described (29). A sandwich ELISA was performed on the culture supernatants to determine the concentration of total IFN-γ. In brief, microtiter plates were coated with primary IFN-γ antibody, blocked with 2% skim milk, and incubated overnight with prediluted supernatants. IFN-γ was detected with a secondary IFN-γ antibody, followed by a horseradish peroxidase (HRP)-conjugated antibody, and the reaction was developed using 3,3′,5,5′-tetramethylbenzidine (TMB) substrate (TMB Plus; Kementec). Plates were read at 450 nm with 620-nm background correction using an ELISA reader (Tecan Sunrise).

### Statistical analyses.

Cells from the murine studies were analyzed using a BD LRSFortessa or BD LRSFortessa X20, and the forward scatter (FSC) files were afterwards manually gated with FlowJo v10 (Tree Star). The graphical visualizations were done using GraphPad Prism v8.3.0. The type of statistical test performed together with the exact *P* value is indicated in the individual figure legends. A *P* value below 0.05 was considered significant.

### Data availability.

The data that support the findings of this study are available on request from the corresponding author.
