# Supplementary figures and images for: In Vivo Antigen Expression Regulates CD4 T Cell Differentiation and Vaccine Efficacy against Mycobacterium tuberculosis Infection
Source: mBio. 2021 Apr 20;12(2):e00226-21. doi: 10.1128/mBio.00226-21 (PMC8092222; doi:10.1128/mBio.00226-21)

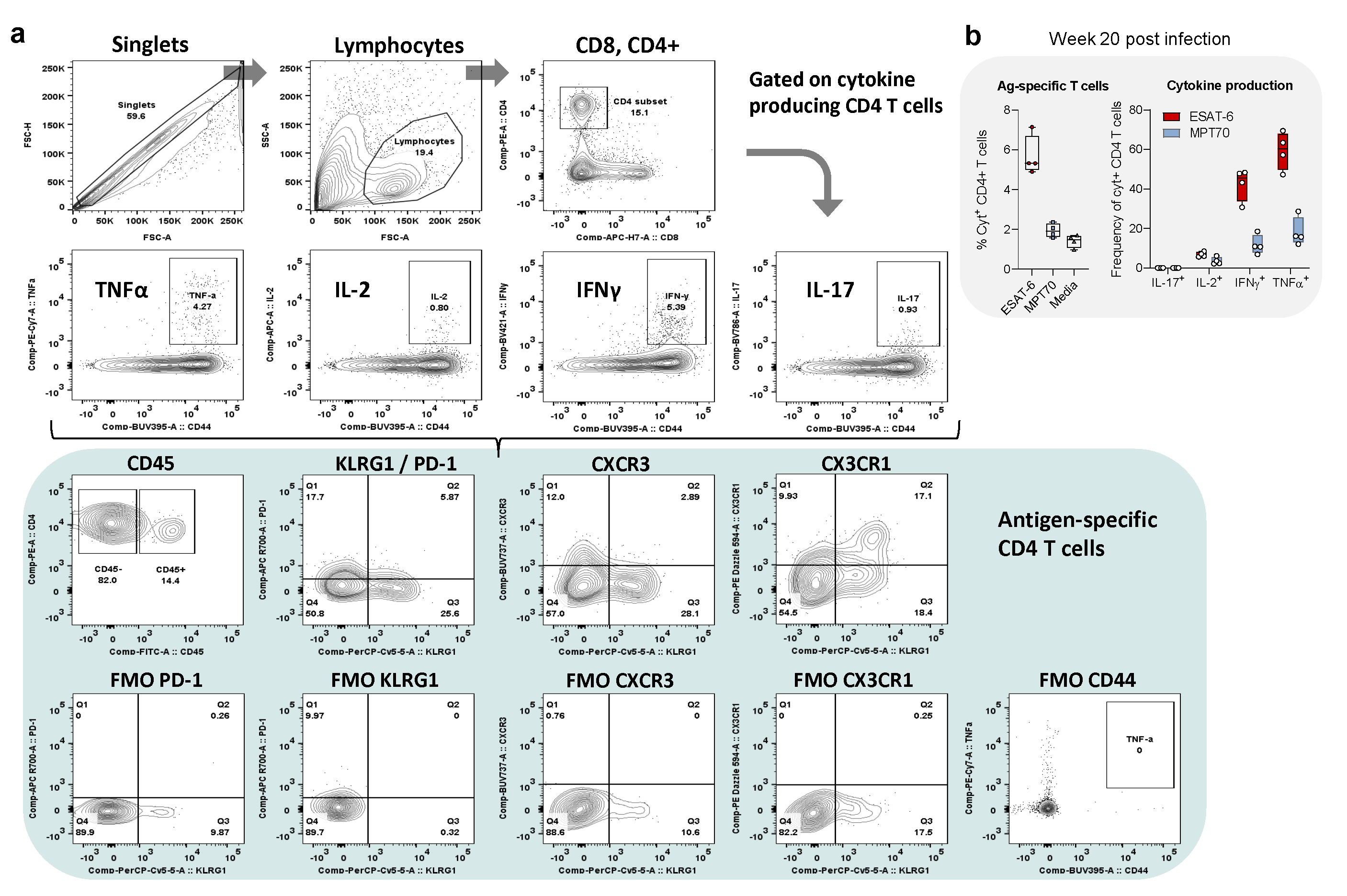

Supplement: FIG S1 [file mBio.00226-21-sf001.tif]

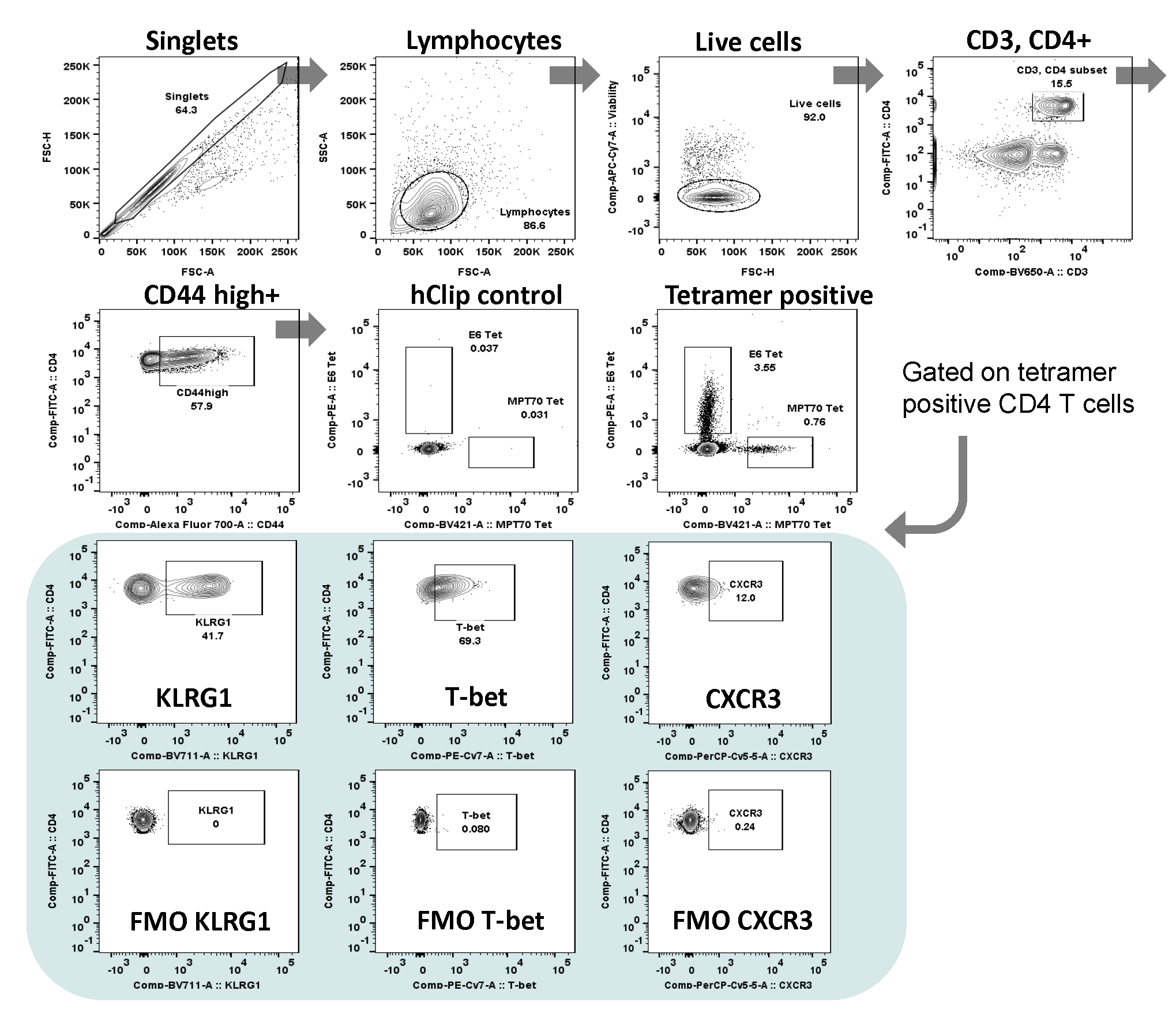

Supplement: FIG S3 [file mBio.00226-21-sf003.tif]

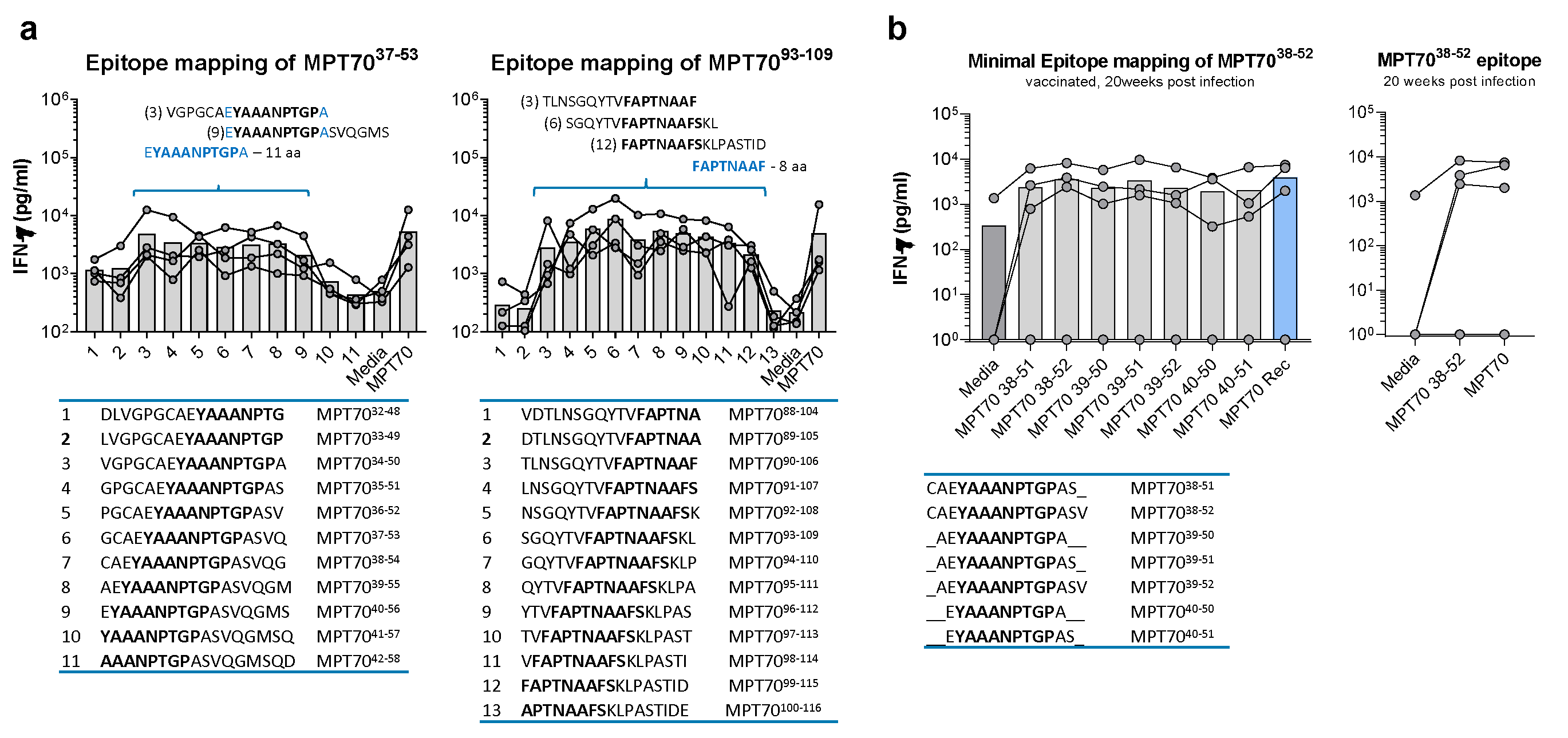

Supplement: FIG S2 [file mBio.00226-21-sf002.tif]

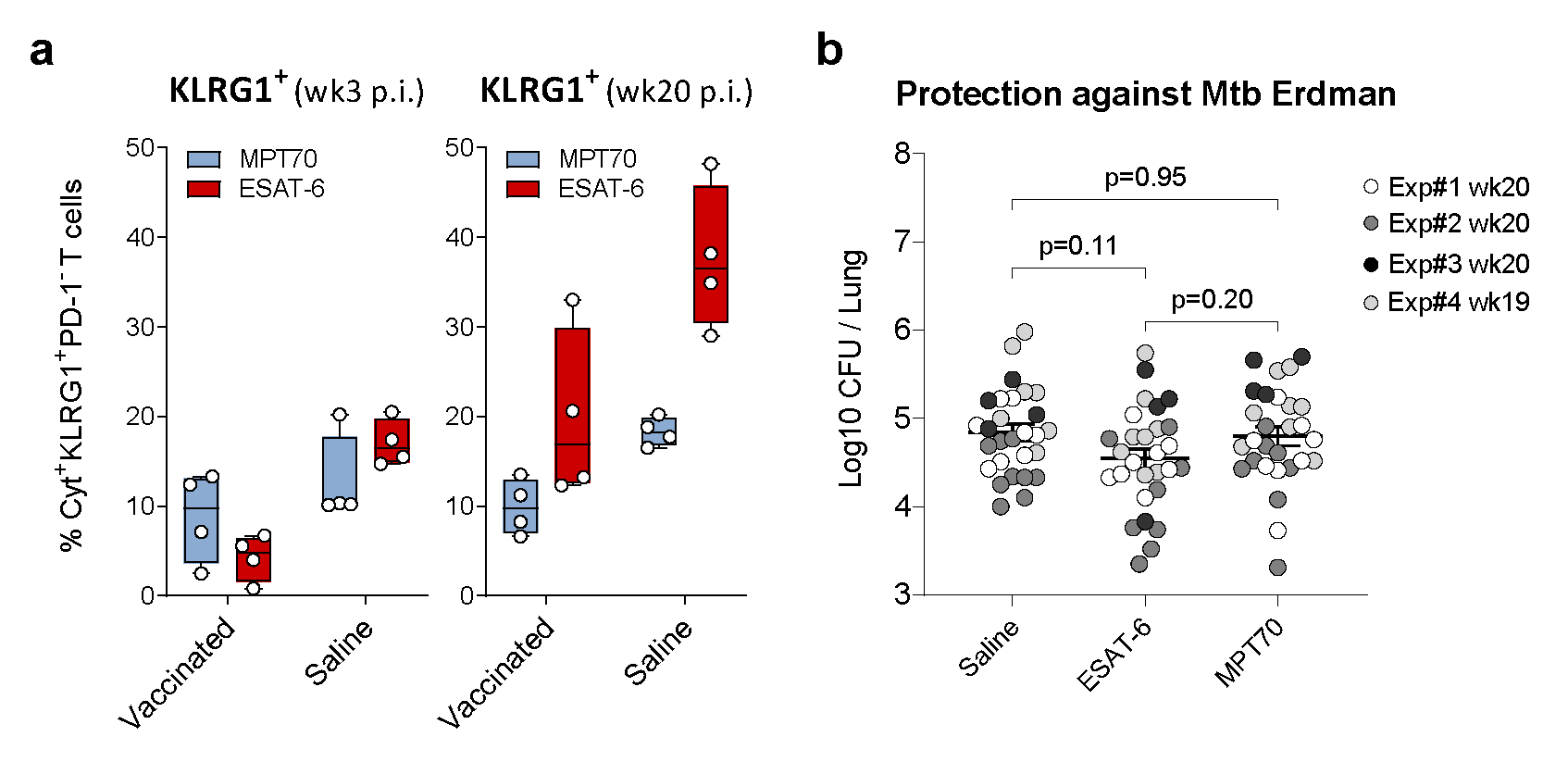

Supplement: FIG S4 [file mBio.00226-21-sf004.tif]

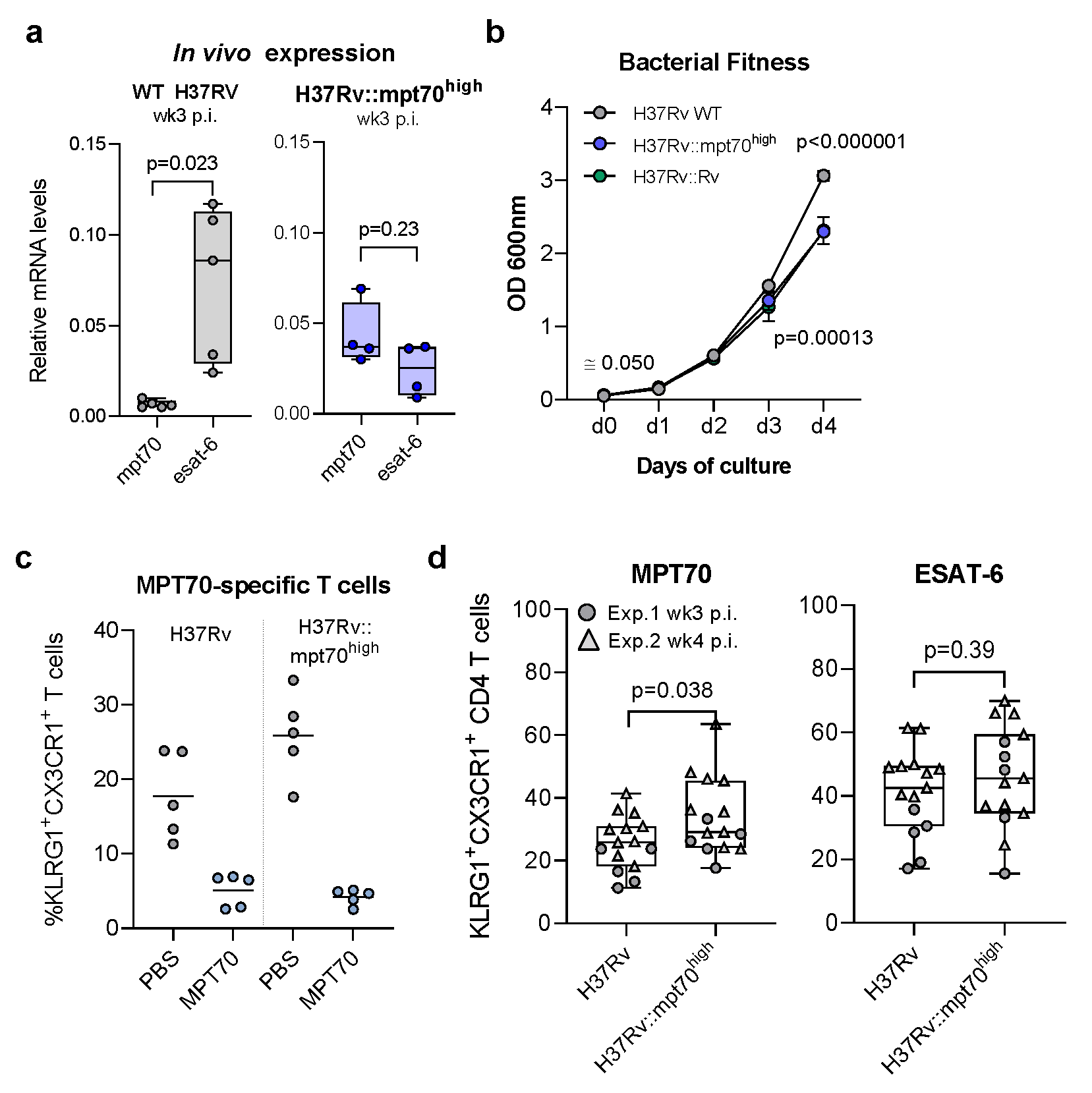

Supplement: FIG S5 [file mBio.00226-21-sf005.tif]
